# The active construction of past episodes

**DOI:** 10.1515/tnsci-2025-0391

**Published:** 2026-03-06

**Authors:** Thomas Parr, Giovanni Pezzulo, Karl J. Friston

**Affiliations:** Nuffield Department of Clinical Neurosciences, University of Oxford, Oxford, UK; Institute of Cognitive Sciences and Technologies, National Research Council, Rome, Italy; Wellcome Centre for Human Neuroimaging, University College London, London, UK

**Keywords:** episodic memory, generative model, active inference, hierarchical, hippocampus, replay

## Abstract

Episodic memories – declarative memories of past events, characterized by rich spatiotemporal context – play a central role in guiding perception and behaviour. Here, we advance a model that integrates episodic memories within the active inference framework. We describe how episodic memories are incorporated into the generative models used in active inference to support the re-construction, replay and communication of past events. In doing so, we foreground two foundational themes. The first is the message passing in deep temporal models that allow one to actively construct memories of episodes. The second is the communicative aspect of declarative memories, and the way in which one might recount something from one’s autobiography. In effect, this means that the message passing that supports episodic memory propagates information about what we have done – or what we would do – given past circumstances to draw inferences about how to communicate those beliefs. Together, these themes emphasise that we are not passive recorders of the things that happen to us. We are active participants in the events we recall and in the telling of stories about them.

## Introduction

With ageing populations globally [1] – and an increasing prevalence of age-related conditions like Alzheimer’s disease [2] that affect memory – an understanding of episodic memory and its computational basis is increasingly important. This may be particularly significant given the promise of disease modifying agents for neurodegenerative pathologies [3], 4]. Both the selection of candidates for clinical trials and treatment, and the measurement of their response to treatment, depend upon careful phenotyping [5]. While much of the focus has been on serum and cerebrospinal fluid biomarkers [6], [7], [8], it is also important to develop an understanding of (heterogeneous) functional deficits in terms of their underlying computational mechanisms. For this reason, we will unpack an approach to modelling episodic memory that lets us examine several computational lesions leading to memory deficits.

Taxonomies of mnemonic processing [9] often begin with the distinction between declarative and non-declarative memories [10]. Simply put, the former are those that can be declared or articulated. Typically, such memories are further divided into episodic or semantic (and sometimes working [11]) memories, depending upon the level of spatiotemporal context. Episodic memories are, by definition, declarative memories with spatiotemporal context. Such definitions are important in that they tell us a great deal about the form of the models that can support them.

From the perspective of active inference [12], behaviour is accounted for in terms of the internal generative world model a brain uses to guide inference (by synaptic message passing) and action [13], 14]. The core idea is that, by predicting the sensory data one would expect if all were going well, our nervous systems can select the actions that realise those predictions [15], 16]. Framed in this way, the definition above tells us two things. A generative model for a creature capable of declarative memories must include a communicative component. If the requisite declarations are verbal, it would need to predict the sensory (e.g., auditory) consequences of speech [17]. For it to be episodic, it must entail statistics about the place and time [18], 19] at which an event occurred.

Memory in natural creatures is a constructive process [20], [21], [22] – not an exhaustive recording of all that has happened in the past. During encoding of memories, a compressed representation is formed that can subsequently be used for reconstruction of the event during recall. However, this poses a problem. If we draw inferences based upon a generative model, how do we go about reconstructing the inferences we made in the past when trying to explain current sensory data? This tells us that a further aspect of memory is the ability to transiently suppress the influence of current sensory data – i.e., to attenuate their precision – during the recall of a memory. Otherwise, the constraints imposed by the world, as it currently is, make it difficult to entertain inferences about the world as it was at some point in the past.

In this paper, we revisit the structure of a generative model previously proposed to deal with the problems of explanation and insight [23]. The ensuing model incorporates both the retention of information about a brief episode of behaviour and the ability to communicate details about inferences drawn during this behaviour. We will use this model as a template to address the way in which temporally deep hierarchies can be used to compress episodes that can later be reconstructed.

In what follows, we start by reviewing the structure of deep temporal models that support inference over multiple timescales [24], 25]. As part of this, we emphasise the concept of precision (a measure of confidence – inverse to uncertainty) [26], [27], [28], which may have relevance both for understanding pathology and therapeutics in amnestic disorders. This somewhat abstract treatment is then applied to a more specific example, in which one must model both an episode of behaviour and the semantic and syntactic states that facilitate questions and answers about the sequence of events in that episode. This minimal example offers a computational taxonomy that can be derived by examining the effect of different kinds of lesions. In the discussion, we then turn back to the question of action in memory and ask what sort of predictions one might make about the neurobiology of synaptic message passing under different generative model architectures. We will suggest that a core difference between isocortical (6-layered) and allocortical (3-layered) cytoarchitectures [29] lies in their relationship to the prediction of the consequences of pursuing a policy or of predicting the (compressed) policy itself, respectively.

## Deep temporal models and episodic compression

**Figure 1:**
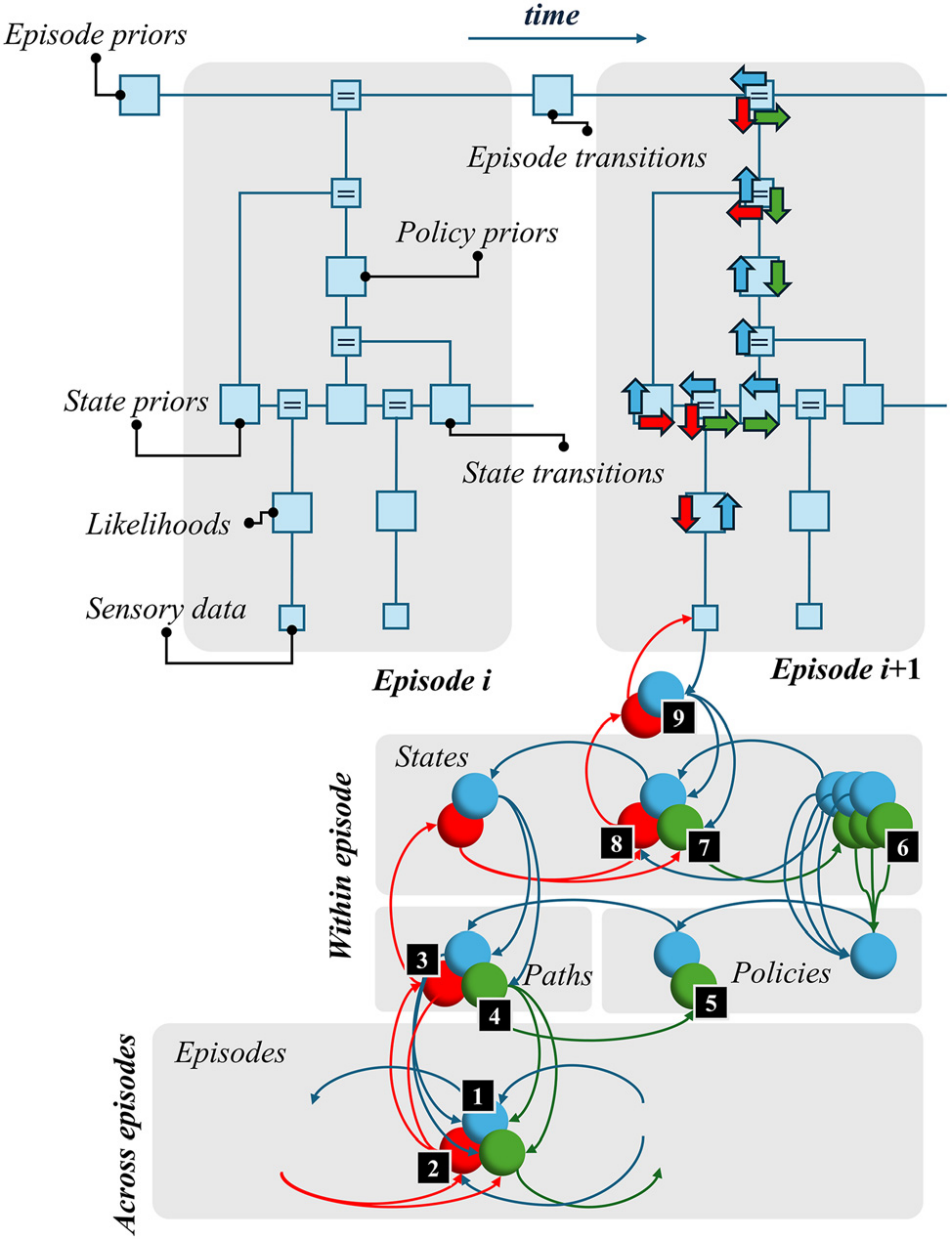
Deep temporal models and message passing. The upper part of this figure expresses a deep temporal model as a Forney factor graph. Each square represents a probability distribution. The lines (‘edges’) connecting those squares are the variables shared by different distributions. Squares containing equals (‘=’) signs enforce equality of all connected edges. For the first episode, the distributions are annotated as prior (including empirical priors) beliefs, transition probabilities, and likelihoods that give the probability of sensory data given some state of the world. The second episode is equipped with arrows that represent the process of inference – here assuming a belief-propagation like scheme [35]. The red arrows show the ‘descending’ messages that predict sensory data. The blue arrows show ‘ascending’ messages that communicate the evidence from those data. Green arrows deal with predictions through time. The lower part of the figure reinterprets these arrows as neuronal populations, preserving the colour coding. We have not reproduced the entire network that would solve the model above – just the portion for which arrows are shown in the upper figure. This is the reason for the connections to populations not shown in the ‘across episodes’ panel. The numbering in black squares is for reference in a later figure, and we will not go through these in detail here except to say that population #6 follows a slightly different pattern to the other messages. Here, we have a series of (blue and green) populations representing the messages concerning anticipated future states under each alternative policy or plan. These are used to update beliefs about which policy to pursue.

Deep temporal (a.k.a., hierarchical) models capture the notion that one can describe the world as evolving over multiple distinct timescales (see [30], 31] for recent theoretical perspectives on this). While some things change very slowly, or perhaps not at all, others evolve more rapidly. Such models have an inherent episodic quality to them, in that they decompose a timeline into a series of short sequences [24]. This means a state at a higher (slower) level of a deep temporal model predicts the features of an episode unfolding at the (faster) level below.1In what follows, we use the term ‘episode’ to indicate the sequence of ‘events’ that play out during an epoch of time that comprises multiple discrete time-steps. This is illustrated graphically in Figure 1, which shows a factor graph representation of a deep temporal model. Each state at a higher level predicts the initial state and transition probabilities for the level below – i.e., it predicts a short trajectory or sequence. Figure 1 uses two kinds of graphical representation. The upper part uses a Forney factor graph notation [32], 33] to describe the elements of an internal model our brains might use to predict sensory data. The lower part illustrates the synaptic message passing between different neuronal populations under the assumption that our brains solve this internal model using a form of belief propagation [34], 35]. Details are unpacked in the figure legend.

Each episodic state, at the highest level, predicts the initial state and policy that, together, imply a short trajectory (i.e., sequence or path) at the level below. In this sense, episodes are compressed representations of a sequence of events. This is analogous to the idea that a sentence is a compressed representation of a sequence of words, and a word is a compressed representation of a sequence of letters. One can imagine generating data starting from the upper left of the factor graph of Figure 1: first sampling an episode, then the initial state and policy expected for that episode, the sequence of states these imply, and finally the sensory data these states generate. To infer the causes of those data, our brains must undo the steps the world has taken to generate them. In other words, the neuronal networks that invert a generative model must have a structure that mirrors the generative model. This is often understood in terms of message passing [36], [37], [38], [39], and illustrated in Figure 1 with a set of arrows that indicate the messages propagated across each factor of the model. Each of these arrows is represented by a node in a neuronal network in the lower part of the figure. This gives us a sense of the kind of computational architecture required to solve temporally structured inference problems.

## A generative model for episodes

**Figure 2:**
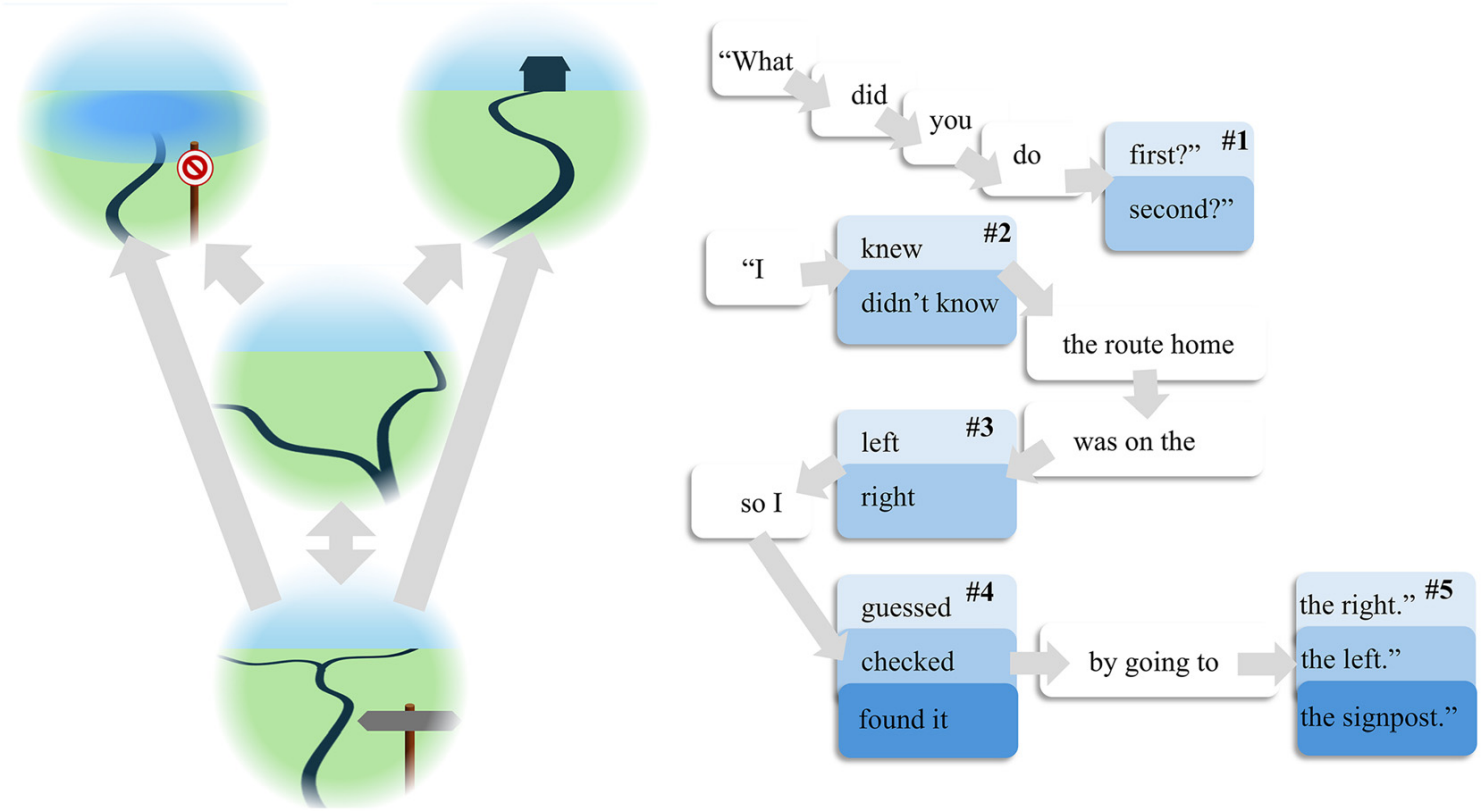
Within episode generative model. This graphic illustrates the structure of the state-space available within a single episode. On the left, we see a set of states that can be occupied during the walk. Participants start at the junction (middle) and from here can either return to the signpost or select the left or right path. On the right, we show the available syntactic and semantic states for verbal communication. These make up a limited language comprising a small number of possible sentences. The initial state for each epoch is either a ‘no speech’ state, the ‘what’ state, or the ‘I’ state. The latter two progress through a defined sequence, with certain points at which alternative words can be substituted. The # states are the semantic states, and the alternative values each of these can take is shown in different shades of blue. The #3 state also doubles as a state for the behavioural task on the left, and is expected to generate both the context (left or right) determining whether the path home is to the left or right, and the spoken word in the corresponding syntax.

The paradigm we model is predicated upon the idea that the simplest way to assess episodic memory is to ask someone questions about something that has happened to them. In unpacking this, it is useful to have a toy scenario in which different episodes could have played out. Let’s imagine a scenario in which our synthetic interviewee is out walking an unfamiliar route and reaches a ‘Y’ junction with two possible paths ahead. They know that one of these paths will take them home, while the other path is flooded ahead and should be avoided. However, not being familiar with the route, they are not sure which is which. They recall having seen a signpost shortly before, which might be informative about the route. They have the option to go back to the signpost to check, or to commit to one of the routes ahead and find out – after walking for another couple of hours – whether they have made it home or not. The left panel of Figure 2 sets out the structure of this task, with arrows indicating the transitions one can make between the different states.

This set-up is formally identical to the T-maze task that has been used as a benchmark for active inference schemes [40], simply capturing a mix of exploitative and explorative behaviour. As in [23], we build upon this by allowing for a subsequent epoch of time in which the person is first asked a question about what happened, and then given time to answer this question. To report on what happened during the walk, it is necessary to maintain a (possibly compressed) representation of the events that took place and to translate these into natural language. This means the memory is both episodic – in the sense that it has spatiotemporal structure – and declarative. The right panel of Figure 2 shows the set of linguistic states in a simple language model that supports queries and answers about the walk.

Recall the generative model in Figure 1 entails conditional probability distributions that express the relationships (squares) between states (edges). The states shown in Figure 2 map to the within episode elements (edges) of Figure 1. The arrows between states in Figure 2 represent possible transitions that are shown in the factor graph of Figure 1 by the square labelled ‘state transitions’. The edge coming the left of this square represents the state (e.g., the first syntactic state) that precedes the state (e.g., the second syntactic state) represented by the line on the right of the square. In short, Figure 1 details the structure of the probability distributions (and neuronal message passing) while Figure 2 tells us about the states that are described by those – within-episode – distributions (and messages).

**Figure 3:**
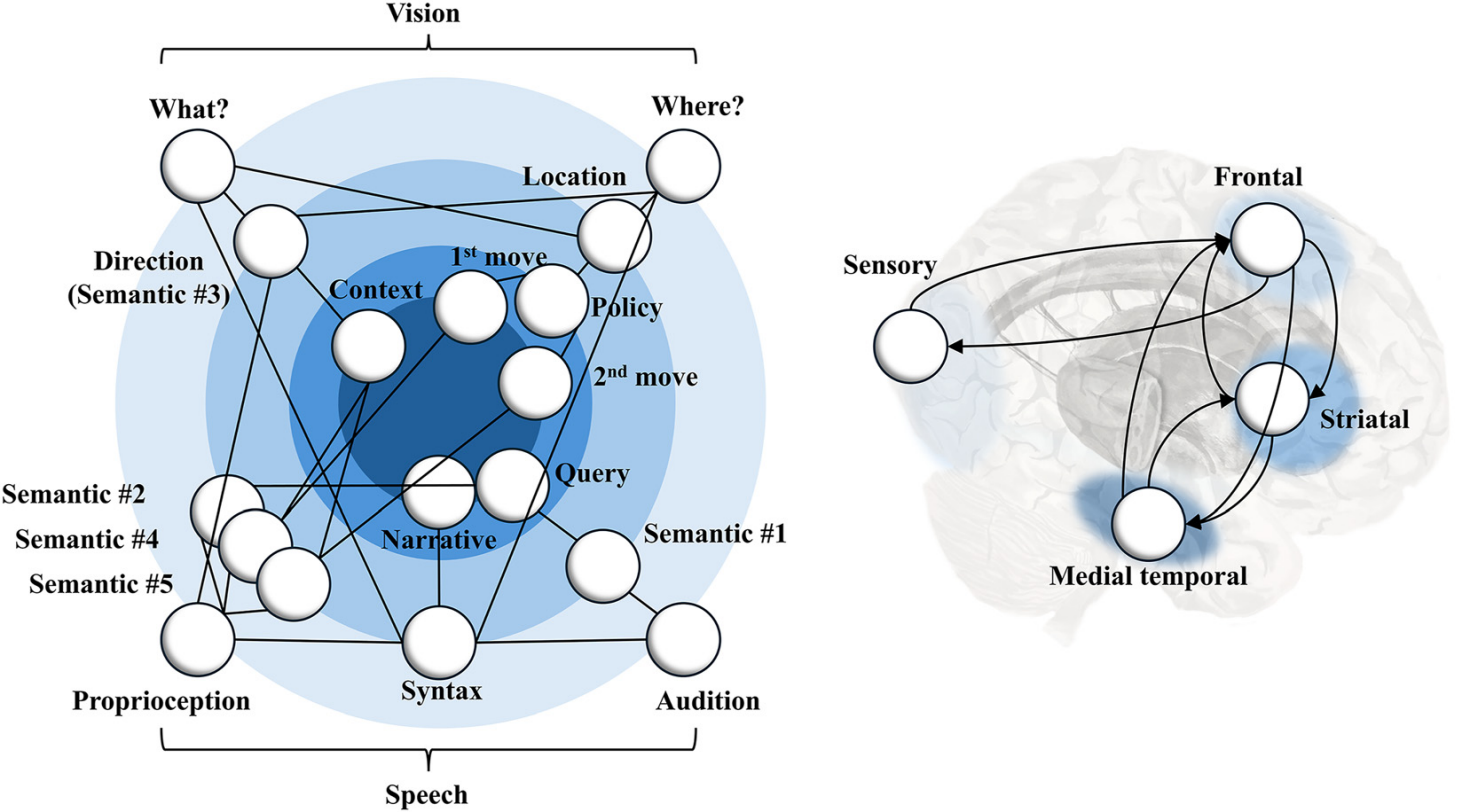
Depth and factorisation. The graphic on the left shows two kinds of factorisation and the variables in play. This is a non-standard graph notation that seeks to complement the information present in Figure 1. Here, we collapse over the temporal dimensions shown in Figure 1 in favour of an expansion of the factorial dimensions (some of which were unpacked in Figure 2). It can be read analogously to a Bayes net, but where the outer variables are conditioned upon the variables closer to the centre of the graph. Here, the radial dimension corresponds to the hierarchical level, getting faster (and closer to sensory data), while the circumferential dimension shows the factorisation of different kinds of states or sensory modalities. The edges (lines) indicate the conditional dependencies between the different states mediated through conditional (empirical) priors and likelihoods. On the right, a loose relationship is shown between different timescales (matching the colours on the left) and representative brain areas that might operate at episodic state (medial temporal), policy (striatal), fast state (frontal), and sensory (occipital) timescales. The arrows here indicate the reciprocal message passing (see Figure 1) expected between these regions.

The left panel of Figure 3 sets out the relationship between the variables of Figure 2 and slower (episodic) variables. This is structured such that the sensory modalities are represented in the outer ring, and the slowly changing variables that offer a minimal (compressed) representation of an episode populate the inner ring. The separation into different types of variables within each ring speaks to a ‘horizontal’ (as opposed to the ‘vertical’ temporal) factorisation of the generative model [41]. From this graphic, it is evident that beliefs about the sequence of locations visited in each episode are informative for beliefs about the policy chosen, themselves informative for beliefs about the 1st and 2nd moves made. In subsequent episodes, this means beliefs about the 1st and 2nd moves constrain the semantic states, themselves constraining the predicted proprioceptive and auditory consequences of speech. In doing so, this allows information from one episode to be propagated into a future episode in which one answers questions about past events.

An important element of the connections shown here is that the ‘syntax’ state connects to all four sensory modalities. The reason for this is that when a verbal syntax is in play (i.e., when speaking or listening) we expect the visual setup to be static. When there is no syntax in play, we do not expect speech. This is a subtle but important point and means that there is an effective suppression or attenuation of some sensory modalities during behaviour, and others during recollection. In effect, this means that a functional disconnection, during recall, liberates belief-updating from the constraints imposed by current sensory data. This allows for reconstruction of past events based upon episodic prior beliefs.

The graphic on the right of Figure 3 offers a simplified representation of the anatomical structures we might expect to be involved across several of these hierarchical levels. Sensory, including visual, regions in the posterior cortices interact with higher cortical areas. These might include regions of the frontal (or perhaps temporoparietal) cortices. We might expect the latter to deal with beliefs about relatively quickly changing, controllable, variables. These could be contextualised by beliefs about slowly changing episodes of the sort associated with medial temporal lobe structures. Both cortical regions interact (directly or indirectly) with subcortical structures, such as the basal ganglia involved in setting trajectories of actions [42].

## Simulating beliefs and behaviour

**Figure 4:**
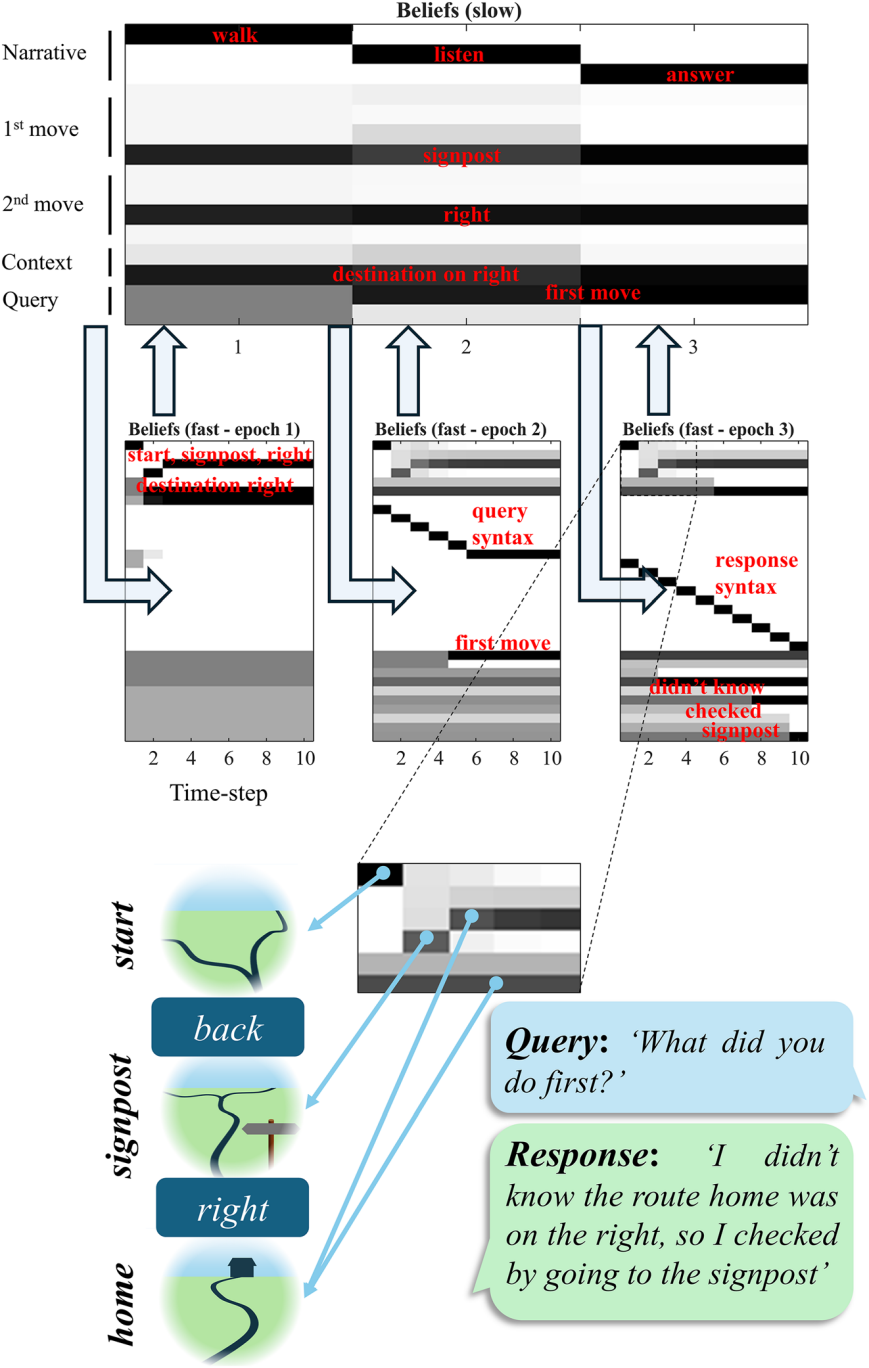
The structure of a simulation. This figure shows how a simulation of belief-updating and behaviour can be visualised. The second level (episodic) beliefs are shown for each of the three epochs (columns). These are shown as the probability (1=black, 0=white) of each state as computed at the end of the epoch (after the fast level below has finished updating). The within epoch states are shown below in detail. Each of these has 10 time-steps. All state factors are concatenated vertically. For ease of interpretation, the sequence of inferences is annotated in red. Below, the replay in the final epoch is enlarged and annotated with graphics to show the remembered sequence of events and behaviour. The query in from the second epoch is shown below this, and finally the response given in the third epoch.

By passing messages, as indicated in Figure 1, between nodes of the generative model, we can simulate the behaviour and belief-updating that results from interaction with a simple synthetic environment. Figure 4 shows an example of ‘healthy’ simulation, to illustrate the format of subsequent simulations. This closely follows the format of the template in [23], and is unpacked in detail in the figure legend. In short, the graphic in the figure shows the posterior beliefs held by a participant at the end of each time-step (or epoch, for the slower level). The rows each represent alternative states. These are concatenated across different state factors. To avoid visual clutter, only those states that are inferred to be most probable are annotated, with labels omitted from those deemed to be implausible. The full set of possible (within-episode or fast) states is given in Figure 2. During the simulation, actions are taken between each time-step, resulting in a new observation at the next time-step. At the slow level, the overall narrative plays out based upon a prior belief that there will be a sequential transition from the ‘walk’ to the ‘listen’ to the ‘answer’ states. In addition, the slow level includes time-invariant states encoding a compressed representation of an episode (first and second moves and other contextual information) that are inferred as more information becomes available.

The sequence of events during the simulation are seen below. During the first episode, our subject starts at the junction. They then move back to the signpost, to determine which direction is home, before then taking the path to the right to reach home. They are then asked about the first move they make, and explain that, as they were not sure which route to take, they returned to the signpost to find out. This demonstrates a minimal sort of behaviour consistent with episodic recall, involving a reconstruction of previous events from a compressed representation that can be used to declare what had happened.

Because this behaviour depends upon simulated message passing, we can interrogate the beliefs implied by those messages. At the slower level or scale we see a progression through episodes involving walking, listening, and then answering questions. During the first episode – of actively engaging with the environment – our synthetic agent draws inferences about their actions, and about the context (whether the destination is to the left or right). These beliefs underwrite action selection and are propagated – as precise posterior beliefs – through to subsequent episodes. The nature of the query is inferred during the second episode, and the ensuing posterior beliefs furnish everything that is needed to answer a query in the third episode.

A more detailed account of the events within each episode is evident in the faster scale inferences below. Each episode involves 10 time-steps – long enough to capture the longest of the syntaxes in Figure 2 in a single episode. As we noted in [23], an interesting feature of this formulation is that there is a kind of replay [43], 44] evident at the start of each of the faster episodes. This is shown in greater detail in the enlarged section of the plot shown below, which is annotated to show the implied beliefs. The reason the inferred behaviour is replayed in both the second and third episodes, despite the behaviour (and its sensory consequences) not being repeated, is because these episodes are associated with syntaxes that decouple the visual modalities from their causes (c.f.: closing one’s eyes to recall a recent episode). In the absence of informative sensory data, the sequence of events depends upon the empirical prior beliefs communicated from the slower level, which reconstructs (i.e., replays) the events from the first episode [45].

**Figure 5:**
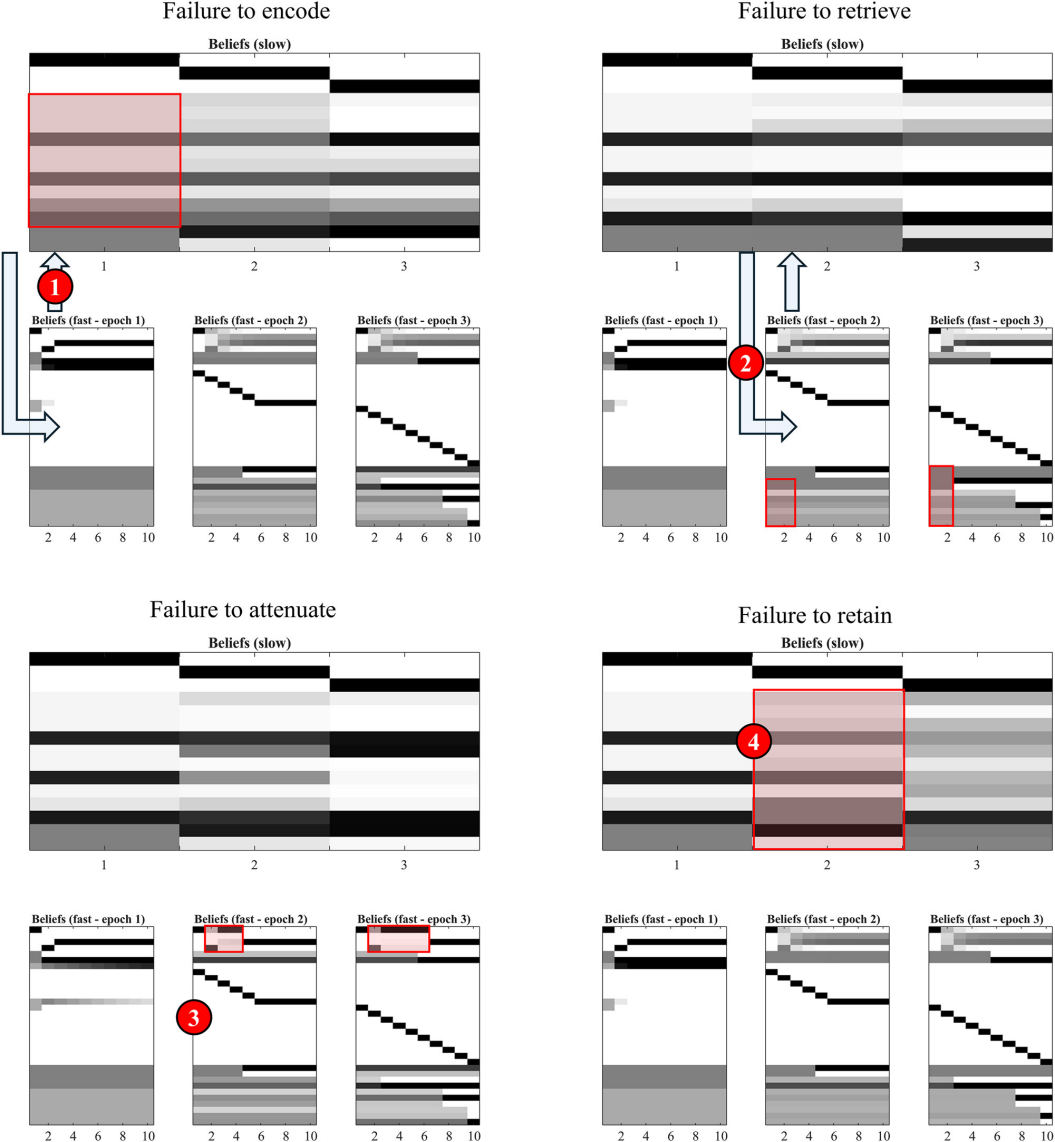
Computational neuropsychology. Here, we present 4 simulations that each deal with a different kind of lesion to the inference process. The format of each is the same as the annotated example in Figure 4. These offer a means to formalise the effect of lesions to parts of a generative model to develop a computationally informed taxonomy of amnestic patterns. The key differences compared to Figure 4 are highlighted in red boxes, with the red circles highlighting where the underlying message passing is disrupted. For example, the attenuated precision with which the location states are predicted in the upper left means that messages passed back up to the second level after behaviour are less informative (#1), leading to imprecise updating of beliefs at the higher level (red box). Analogous lesions are shown in the other panels as described in the main text.

In Figure 5, we demonstrate the undesired outcomes or impairments resulting from these lesions. Specifically, we simulate four distinct computational lesions. These are:

– Failure to encode – reduced precision in the predictions2Technically, the word ‘prediction’ here refers to either a posterior predictive density (where it is applied to sensory data) or to an empirical prior (where it is applied to hidden variables such as policies or initial states). Given a conditional probability distribution *P*(*y*|*x*), and beliefs about *x*, *Q*(*x*) (which may be a prior or a posterior depending upon past data) a ‘prediction’ one might make about *y* is the expectation (average) of *P*(*y*|*x*) under the distribution *Q*(*x*). from the slow to the fast level for initial location, context, and policy states.
– Failure to retrieve – reduced precision in the predictions from the slow to the fast level for semantic states.
– Failure to attenuate – impaired suppression of predictions of visual outcomes during listening and answer episodes.
– Failure to retain – reduced precision for transitions at the slower level (i.e., loss of information between episodes)

Taking these in turn, the failure to encode – shown in the upper left of Figure 5 – depends upon the generative model having an imprecise mapping between the two levels of the hierarchical model. This means that reciprocal message passing between layers, particularly during the first epoch, is attenuated. As a result, the updating of the slower states, following inference about the faster states, leads to a less precise set of beliefs. This has implications for the beliefs propagated to subsequent epochs. Note that, in the third epoch, precise beliefs about the context are recovered at step 6. This is the point at which our participant hears themselves saying ‘right’, emphasising the fact that telling the story can influence our memory of it (in this case, correctly).

The failure to retrieve is very similar in nature – affecting the precision of messages between levels – but affects the prediction of semantic states at the fast level from the episodic states at the slower level. This has two consequences. The first is that, in the second and third epochs, the semantic states are initialised with much greater uncertainty, despite the memory having been formed with relatively high precision at the second level. Second, the query posed is imprecisely passed up to the slow level from the second epoch. Although the replay of the episode is accurate, the construction of a veridical answer to the question is compromised. In fact, the answer given here is the correct answer to an unasked question about the second move (‘*I knew the route home was on the right, so I found it by going to the right*’). However, one could imagine that extreme versions of this disconnection could lead to semantic aphasia syndromes of the sort that can be seen in both Alzheimer’s and Frontotemporal dementias [46], in which grammatical structure is preserved but the ability to express meaningful speech is lost.

The failure to attenuate is particularly interesting in that it compromises the replay, but with relative preservation of the semantic states. To recap, the attenuation present in the healthy simulation results from the form of the conditional distribution mapping fast states to visual outcomes. The states upon which this is conditioned include the syntactic states which set a precise relationship between location and visual states when there is no speech present, but an imprecise mapping when the syntactic states are consistent with the question or answer syntaxes. In the extreme, this means visual outcomes are (believed to be) informative about location states only when not listening to – or answering – questions. In effect, this means attending away from (i.e., attenuating) vision during engagement with questions. Failure to attenuate suppressed replay during these times. This is because the slower (episodic) level has no problem in assimilating the first episode from the lower level and communicating the relevant semantic states to the second and third epochs. However, during the second and third epochs, the visual data (set somewhat arbitrarily to a view of the junction) conflicts with the predictions made by the slow level. When these conflicting observations are not fully suppressed, we see them corrupt the replay – here showing a return to the crossroads after the signpost and not progressing to the final location until later. The same conflict is not relevant for the semantic states, as the auditory (and proprioceptive) speech data are perfectly consistent with the predicted semantic states. This phenotype might reflect a kind of aphantasia [47] in which people struggle to form mental imagery from memories or imagination. In other words, this phenotype is explained by a difficulty in dissociating oneself from the present – something that has been reported in cases of hippocampal amnesia [48].

Finally, when we suppress the precision of transitions at the slow (episodic) level, we reduce the information propagated from one episode to the next. Specifically, those elements of the compressed episode (the identity of the first choice, the second choice, and the true direction home) that are represented as time-invariant states would normally transition to themselves. When the precision associated with these (between epoch) transitions is diminished, information about these states garnered during the first epoch is lost with each subsequent transition. By the second epoch, the empirical priors communicated to the lower level are much less certain. Note that, even with these imprecise beliefs the faster inferences still reproduce at least some of the trajectory. This is because the evaluation of policies at the lower level depends partly upon priors and partly on beliefs about ‘what I would do’. However, this is not sufficient to determine the context (i.e., the route home) as this information has been lost. By the third epoch, even the question has been forgotten. There is a retrospective inference it must have been about the second move based upon this being more consistent with the answer given (‘*I didn’t know the route home was on the right, so I checked by going to the right*’). Going to the right is more consistent with a second move than a first.

## Discussion

To summarise the preceding sections: we used active inference – under a generative world model – to explain sense-making and decision-making. A natural form for the requisite generative model involves a separation of temporal scales, such that sequences of fast events can be summarised in terms of sequences of episodes. In other words, an episode is a compressed representation of a short sequence of events. Episodic memory is a term from psychology with a specific meaning. A core aspect of this meaning is that it is a declarative form of memory. This implies that it is not enough to store, or even replay, a sequence of past events. One must be able to declare or communicate these events at a later point in time. We outlined a generative model capable of generating both sequences of events and the linguistic responses apt for answering questions about those events. Our numerical simulations illustrate the successful performance of a simple sequence involving behaviour, questioning, and answering such questions. Mechanistically, the message passing between hierarchical levels entailed a kind of replay, in which beliefs about previous sequences of events were re-instantiated during question-and-answer phases.

We outlined four possible computational lesions – pathological perturbation of parameters – to examine the accompanying mnemonic phenotypes. These included: (1) the attenuation of precise mappings between the two hierarchical levels that led to a failure to encode, as is often associated with diagnoses of functional cognitive disorders [49]; (2) an attenuation of the precise mapping between levels for the semantic states relevant to explanation, which led to a failure to retrieve stored memories – a deficit thought to be a particularly prominent feature of some forms of mild cognitive impairment [50]; (3) failures to attenuate, or attend away, from current sensory input during recall suppressed the replay effects outlined above – something that may be relevant in both aphantasia and hippocampal amnesias; (4) and finally, failure to retain memories was found to result from attenuation of the precision of higher-level transitions, meaning that states previously believed to be fixed and time invariant were instead treated as volatile – as if the identity of a state in the past was uninformative about that same state at a later time.

The simulations reported above afford a means of validating the message passing set out in Figure 1; in that they demonstrate plausible patterns of activity associated with different lesions of mnemonic processing. However, many models [51], [52], [53] of episodic memory have previously been formulated without appealing to generative modelling. It is reasonable to ask what insights we gain by doing so. Among these are the ability to generate an end-to-end simulation of beliefs and behaviour, which go beyond a proof of principle model of a single brain region. Perhaps the closest to the approach advocated here is that of [54], 55], which deal with generative models of the sort seen in deep learning. A core difference between deep learning and active inference formulations is our focus upon action and the idea that our past behaviour is a core element of our (first person) memories of episodes. Other accounts of memory and hippocampal function that appeal to active inference principles include [56], 57] which provide complementary elements of our treatment. The former emphasises the vertical (hierarchical) structure of the sort we have employed here. The latter emphasises the horizontal factorisation that gives temporal sequences meaningful structure. Both forms of factorisation are essential in the account we offer here.

**Figure 6:**
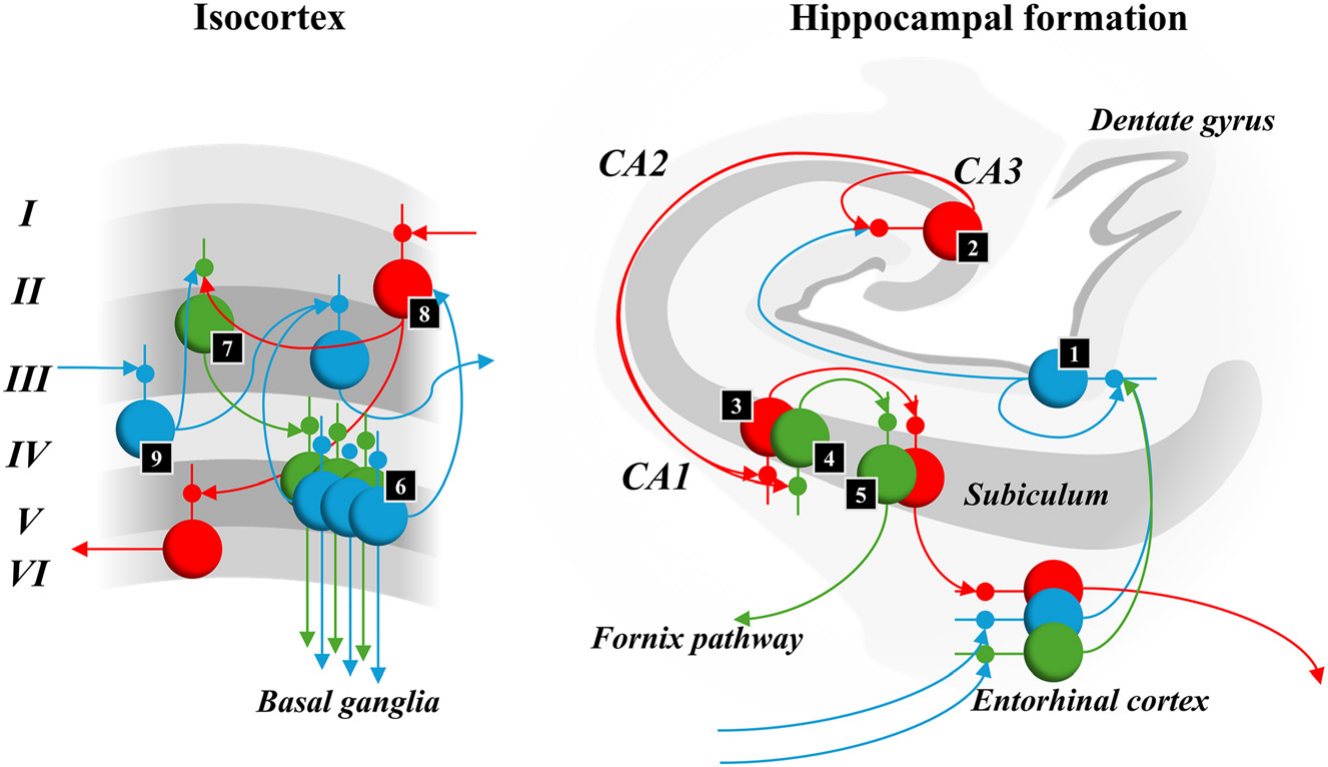
Microcircuits and messages. This figure addresses the finer-scale anatomy of different cortical cytoarchitectures and of the message passing implied by a belief-propagation scheme that does and does not disambiguate between alternative trajectories. The numbers correspond to those in Figure 1. The isocortical layers are shown from superficial (layer I) to deep (layer VI) in the vertical dimension. The hippocampal formation is shown as a coronal slice. For each of these, we preserve the connectivity implied by the model of Figure 1 and the patterns of laminar connectivity known from empirical research as summarised in the main text. The convergence of ascending and descending messages onto population #7 in the isocortex graphic facilitates prediction of several possible alternative futures (populations labelled #6). Perhaps the relative complexity of the 6 layered neocortex is that it predicts futures conditioned upon actions which, communicated to the basal ganglia via layer V, help in adjudication between plans. In contrast, 3 layered allocortex may predict sequences that offer compressed representations, that are only unpacked into predictions of policies and of states conditioned upon those policies through projections either to neocortex or basal ganglia.

A further, more specific, insight is unpacked in Figure 6. This rests upon the key contributions of active inference to the question of episodic memory: namely, the idea that a memory is a kind of constructive inference, which reinstates – and reinterprets – previous experiences despite the behaviour not being repeated, and that action is inseparable from inference in authentic agents. Figure 6 depends upon and understanding of the anatomy of both isocortical structures and the hippocampal formation. The two differ in that the former relies upon a six layered structure, while the latter has only three distinct layers. The microcircuitry of the isocortex has been unpacked in detail in [58], [59], [60], [61], [62]. In summary, ascending pathways target neurons in layer IV. Descending pathways target neurons in superficial layers. Ascending connections originate from layer III well descending pathways originate from layer VI. Layer V is associated with neurons that target either spinal cord motor neurons or subcortical structures like the striatum thought to be involved in the evaluation of alternative action plans. For a useful overview of hippocampal anatomy please see Ref. [63]. In short, much of the input to the hippocampus comes via the entorhinal cortex which projects via the perforant pathway through to the dentate gyrus. From here neurons project first to the CA3, then from here to the CA1, regions of the hippocampus. CA1 pyramidal neurons target the subiculum, which provides outflow from the hippocampal formation either through the fornix pathway or through projections back to the entorhinal cortex.

Electrophysiological [64] and anterograde tract tracing [65] evidence in rodents – and related evidence from primates [66] – supports a direct projection from the subiculum, via the fornix pathway, to the ventral striatum, a key input nucleus of the basal ganglia. Several polysynaptic pathways from nuclei back to the hippocampus have also been described. These include projections from dopaminergic midbrain to the medial temporal lobe [67] that have been proposed to mediate the robust encoding of a memory. Previous active inference process theories have argued that dopamine signals a precision or confidence in policy selection [68] – the implication being that increased dopamine signalling might influence the confidence with which a policy is inferred to have been selected and lead to more confident, precise, message passing from the policy to the episodic level of a model. In other words, one might interpret dopamine as mediating the opposite effect to that of the ‘failure to encode’ lesion from Figure 5. Other pathways might rely upon the inputs to hippocampus via midline thalamic nuclei (such as the nucleus reuniens) [69].

The graphic on the left of Figure 6, illustrates the relationship between belief updating as shown in Figure 1 and the architecture of 6 layered isocortex. The colour coding illustrates the relationship between hierarchical message passing, with blue representing ascending pathways and red representing descending pathways. The green pathways relate to the message passing through time, necessary for the evaluation of alternative courses of action – i.e., for the anticipation of the future consequences of alternative actions. For comparison, the graphic on the right shows how the message passing implied by our generative model might relate to the architecture of the hippocampal formation. This rests upon the assumption that the hippocampus deals with higher level, episodic, beliefs. To anchor the reader to the neuronal message passing illustrated in Figure 1, the numbers in small black squares in both Figures 1 and 6 illustrate the points of contact between the two architectures. The idea this figure seeks to communicate is that, when considering alternative possible futures, and selecting policies that bring about one of these futures, a more complex architecture supported more complex lamina structure – like that seen in isocortical regions – may be necessary. In contrast, if medial temporal lobe projections to the basal ganglia represent a prior distribution over alternative paths, as opposed to the anticipated consequences of following each of those paths, a simpler trilaminar architecture might suffice.

Before concluding, we considering four plausible extensions of this work. First, we have largely ignored the question of neural plasticity. In this sense, the simulations above relate to a relatively short-term form of episodic memory. To extend this to longer term memories, one might equip the priors and conditional probability distributions with conjugate priors of their own such that one can learn the relationships between different kinds of variables. Under the process theories of active inference [70] this corresponds to updating synaptic efficacies; i.e., active learning.

Second, an important feature – of the generative model we have proposed – is the suppression of current sensory data during the process of recall. This makes a key prediction that could be assessed empirically. One would expect that, during recall of some past event, sensory stimuli should have a higher detection threshold or generate smaller evoked responses in electrophysiological studies. Further to this, one might expect that this effect, if present, would negatively correlate with one’s ability to generate mental imagery.

Third, when introducing generative models for events, we did not detail how events are identified and segmented from continuous streams of experience. Various lines of research reveal that the brain identifies events at multiple hierarchical levels, with event boundaries at each level typically corresponding to moments when unexpected events happen, revealing a deep link between (hierarchical) predictive processing and event segmentation [71], [72], [73], [74]. These insights can be incorporated into active inference agents to make them able to autonomously segment continuous streams of experience into hierarchical episodic components, see for example [75].

Fourth, while we only addressed episodic recall – and the replay of experience – during question answering, hippocampal replay is widely assumed to promote memory consolidation, planning and other cognitive functions [45], 74]. For example, self-generated streams of experiences – or generative replay – can help train and consolidate generative models and enable compositional inference [76]. Endowing the model presented in this study with generative replay could allow it to learn vicariously from self-generated experience, in addition to real physical interaction.

In concluding, we make one further observation, each of the lesions we considered can be understood in terms of a confidence or precision parameter. This is interesting in that these parameters are exactly those that have been previously associated with neuromodulatory chemicals including acetylcholine, noradrenaline, dopamine, and serotonin [81], [98], [99], [100], [101] (see Table 1, adapted from [60], which summarises these associations). The reason for making these associations is that the effect of changing precisions in a generative model is to enhance or attenuate the influence beliefs about one variable have on beliefs about others. When interpreted in terms of synaptic message passing, this is like modulating the effective connectivity (i.e., synaptic gain) contextualising interactions among distinct neuronal populations. This is corresponds to multiplicative gain modulation mediated by cholinergic and catecholamine neurotransmitters.

**Table 1:** Association between precision and neurotransmitters.

Neurotransmitter	Precision	Evidence
Acetylcholine	Likelihood	– Presence of presynaptic receptors on thalamocortical afferents [77], 78] – Modulation of gain of visually evoked responses [79], 80] – Changes in effective connectivity with pharmacological manipulations [28] – Modelling of behavioral responses under pharmacological manipulation [81], 82]
Noradrenaline	Transitions	– Maintenance of persistent prefrontal (delay-period) activity (requiring precise transition probabilities) depends upon noradrenaline [83], 84] – Pupillary responses to surprising (i.e. imprecise) sequences [85], [86], [87], [88], [89] – Modelling of behavioral responses under pharmacological manipulation [81]
Dopamine	Policies	– Expressed post-synaptically on striatal medium spiny neurons [90], 91] – Computational fMRI reveals midbrain activity with changes in precision [92] – Modelling of behavioral responses under pharmacological manipulation [81]
Serotonin	Preferences or interoceptive likelihood	– Receptors expressed on layer V pyramidal cells [93], [94], [95] in medial prefrontal cortex – Medial prefrontal cortical regions heavily implicated in interoceptive processing and autonomic regulation [96], 97]

As such there are potential therapeutic implications in amnestic disorders for which drugs affecting these molecules are already in use. Both Alzheimer’s disease and Lewy Body disease show some clinical improvement with cholinesterase inhibitors [102]. Table 1 implies the effect of these medications is likely at the level of the likelihood distribution, implicating either the failure to attenuate (likelihood of sensory observations given states), or the failure to retrieve (likelihood of fast states given slow states), in their action. The role of attenuation of sensory data is of particular interest in Lewy Body disease, in that a core part of the clinical phenotype is a tendency to hallucinate: i.e., a failure to use sensory data to correct self-generated visual imagery. One could interpret this as a pathological sensory attenuation that specifically relates to the profound cholinergic deficit in Lewy Body disease [103]. This idea is unpacked in greater computational detail in [100].

An interesting question both for existing therapies, and for emerging biologic therapies, is which of the deficits in Figure 4 they protect against. This is important in that it might allow clinicians to assess the likely benefits patients may achieve and to balance these against the risk of different therapeutic options. For those therapies still under development, the computational phenotypes of patients with amnestic disorders may afford a useful criterion to select appropriate (sub)populations for clinical trials, overcoming the dilution of measured clinical effects of interventions in populations with highly heterogeneous disease phenotypes [104]. Specifically, if a disease process (or the population with that disease) is highly heterogeneous, the average effect of a therapy across a trial population may be small even if there is a large effect in a subset of people with this diagnosis. A principled means of selecting that subset offers an opportunity to measure a potentially meaningful effect for those people that would otherwise be obscured by heterogeneity in an unselected population.

## Conclusions

We have sought to illustrate how emerging developments in theoretical neurobiology, specifically active inference, might play a role in our understanding of episodic memory. The contribution of active inference here is the idea that memory is an active process in which one constructs imagery of the past based upon empirical prior beliefs about how one acted, or would have acted, in a given context. The definition of declarative memory is inherently active in the sense that it implies the ability to declare or communicate the contents of a memory. This underlines the importance of developing models that capture both the behaviour during the episode to be recollected but also the declarative process of communicating those events. By modelling the communicative part of memory, we move a step closer to a computational account of the measurements taken by clinicians, implicitly, while listening to a patient’s story.
